# Supplementary figures and images for: Toxoplasma GRA15 and GRA24 are important activators of the host innate immune response in the absence of TLR11
Source: PLoS Pathog. 2020 May 26;16(5):e1008586. doi: 10.1371/journal.ppat.1008586 (PMC7274473; doi:10.1371/journal.ppat.1008586)

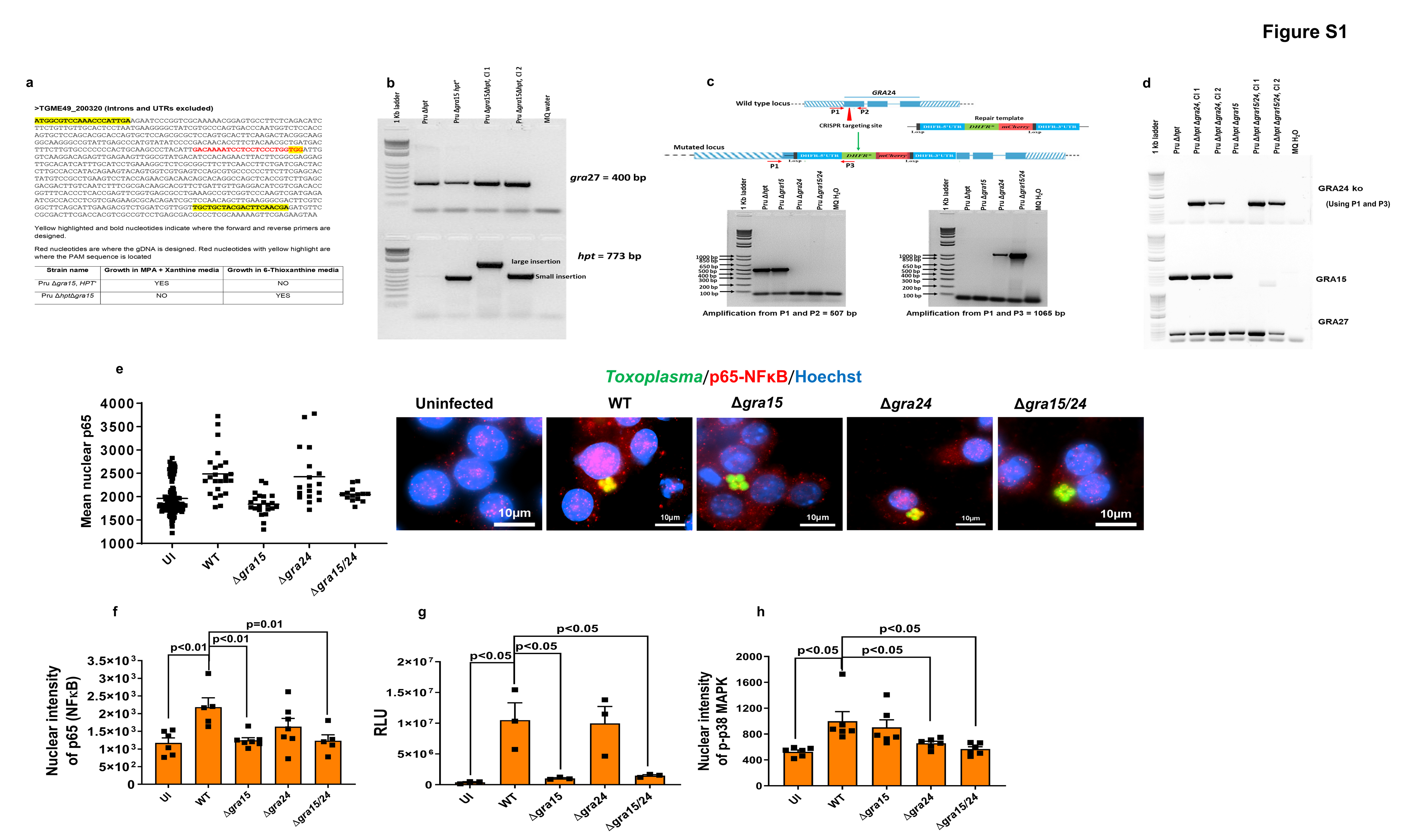

Supplement: S1 Fig — Sequence of the Hypoxanthine-guanine phosphoribosyl transferase (HPT) gene showing the sgDNA sequence in red for Cas9-mediated disruption and primer sequence in yellow (a). Disruption of the HPT gene was determined by using specific primers designed to amplify the region shown in the bottom figure, while GRA27 was used as a housekeeping PCR control (at the top) (b). Schematic diagram of the strategy followed to delete GRA24 (top) and PCR to screen the clones confirming the disruption of the gene (P1+P2) (bottom) is shown in (c). wild-type (left) and the presence of the insertion of the repair template in the locus (P1+P3) (right). Identification of Δgra24 and Δgra15/24 double knockout using specific primer sets for GRA24 (top panel), GRA15 (middle panel) and GRA27 as a control for quality of the input DNA (lower panel) (d). Nuclear translocation of the p65 subunit of NFκB was quantified from infected RAW 264.7 macrophages 18 h p.i. with indicated strains. At least 15 cells were quantified as shown in the graph (left) and representative images are shown on the right (e). Phenotypic confirmation of single clones of wild-type, Δgra15, Δgra24 and Δgra15/24 parasites by their ability to activate NFκB (f,g) and p38 MAPK (h). Each dot represents the mean value of at least 15 host cell nuclei (f and h) or 3 technical replicates (g) from a single experiment. Statistical analysis was done by One-way ANOVA followed by Tukey’s multiple comparison test. Data are represented as mean ± standard error of the mean (SEM). (TIF) [file ppat.1008586.s001.tif]

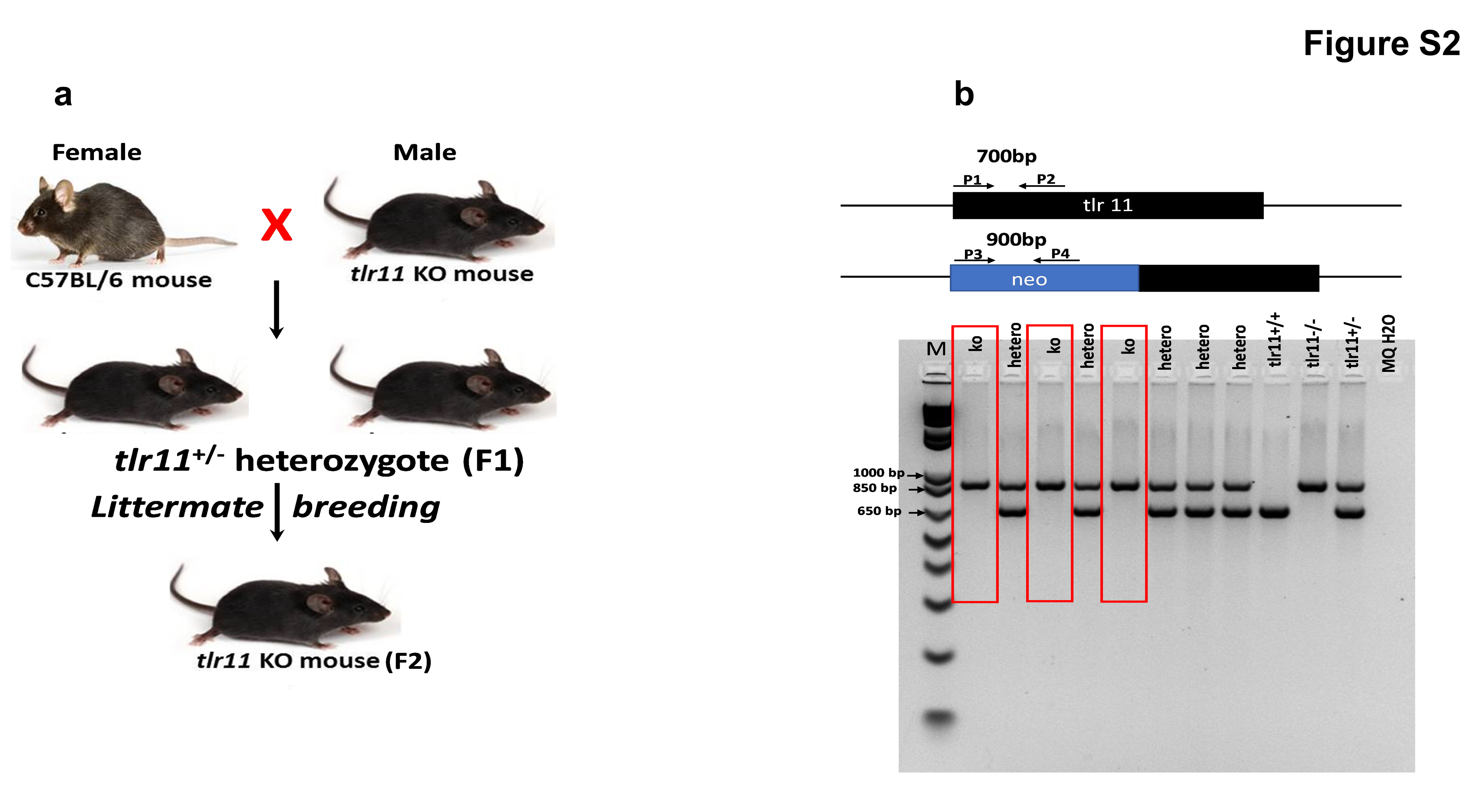

Supplement: S2 Fig — Mouse breeding scheme to generate in house TLR11 knockout mice by cross-breeding homozygous Tlr11-/- male with homozygous Tlr11+/+ female mice (a). Primers in the TLR11 locus (top) and PCR of the F1 progeny (bottom) where the homozygous Tlr11+/+ yields a single band around 700 bp, homozygous Tlr11-/- generates a single band around 900 bp and all heterozygous mice generate both the bands at 700 bp and 900 bp (b). (TIF) [file ppat.1008586.s002.tif]

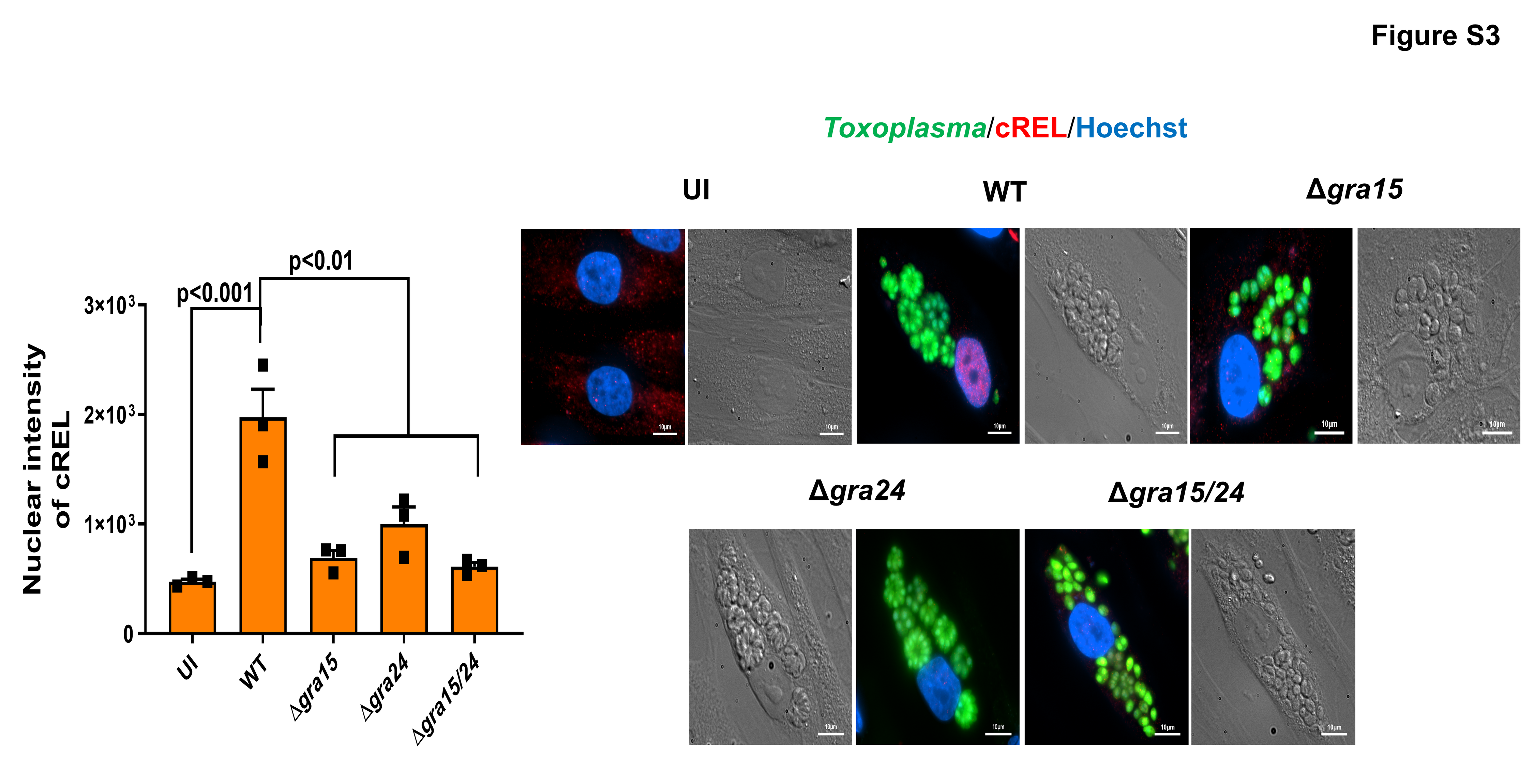

Supplement: S3 Fig — Indicated parasite strains were added (MOI of 3) to confluent monolayers of HFFs grown on coverslips in 24-well plates. 16 h p.i. cells were fixed and stained with cREL antibody. Each dot represents the mean value of at least 15 host cell nuclei from a single experiment. A representative image for each group is shown on the right. Scale bar represents 10 μm. All the data are shown as mean ± SEM. Statistical analysis was done by two sample Student’s t test. (TIF) [file ppat.1008586.s003.tif]

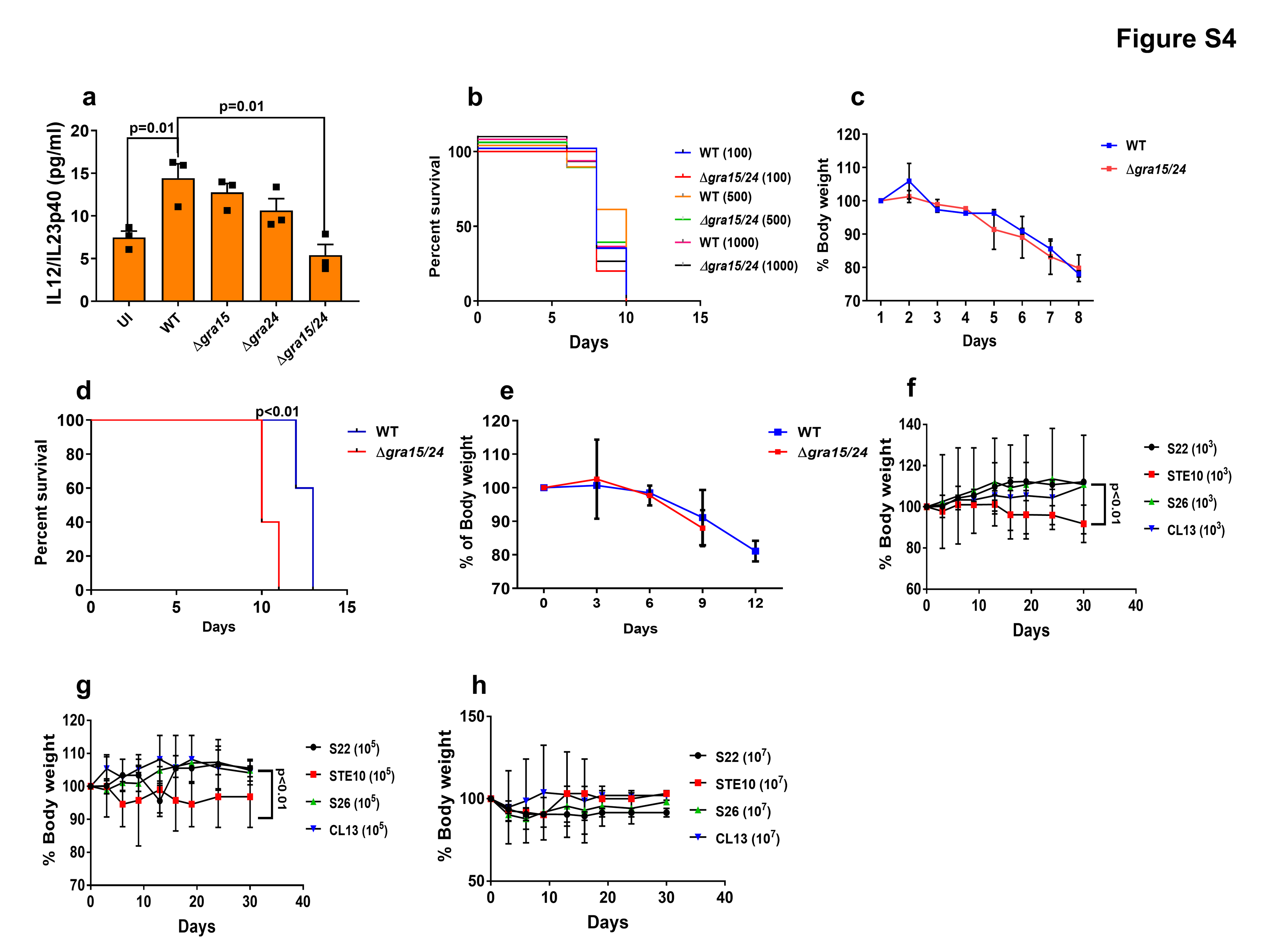

Supplement: S4 Fig — Tlr11-/- mice were i.p infected with 5,000 tachyzoites of indicated Toxoplasma strains and 1-day p.i. serum was collected from each of the groups to measure IL12/IL23p40 (a). Survival and body weight measurements of Tlr11-/- mice (N = 8 mice per group) that were i.p infected with 100–1000 tachyzoites (b-c) or 10 tissue cysts (d-e) of the indicated strains. Tlr11-/- mice were i.p injected with indicated doses of tachyzoites of different Toxoplasma strains derived from F1 progenies of type II X type III crosses (51) and body weight was measured daily throughout the infection (f-h). All the data are represented as mean ± SEM. Statistical analysis was done by two sample Student’s t test and log rank test for survival curve. (TIF) [file ppat.1008586.s004.tif]

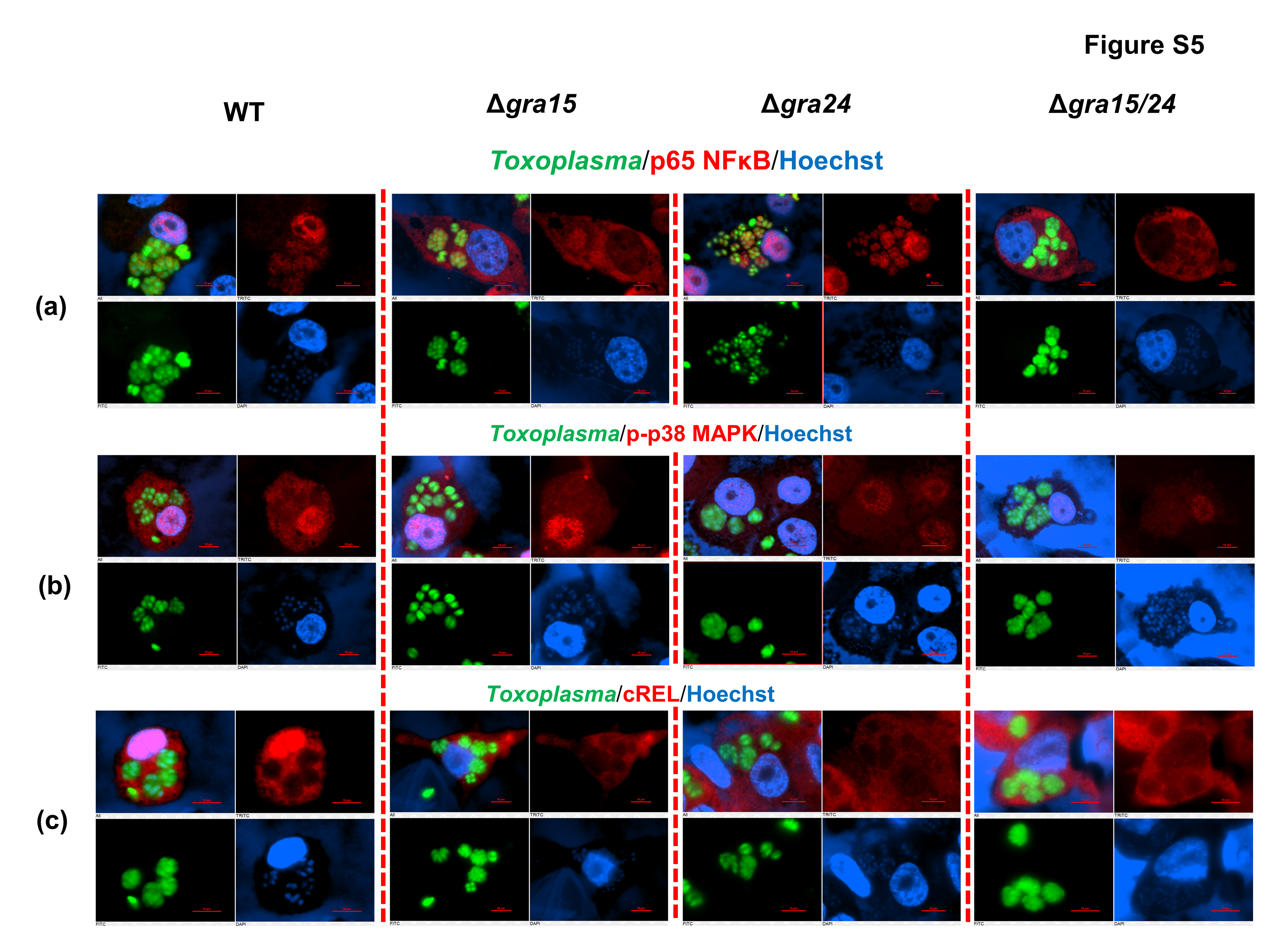

Supplement: S5 Fig — PMA differentiated THP1 macrophages were infected with indicated strains for 24 h and immunofluorescence assay was performed to quantify nuclear translocation of the NFκB p65 subunit (a), p-p38 MAPK (b) and NFκB cREL subunit (c). Scale bar represents 10 μm. (TIF) [file ppat.1008586.s005.tif]

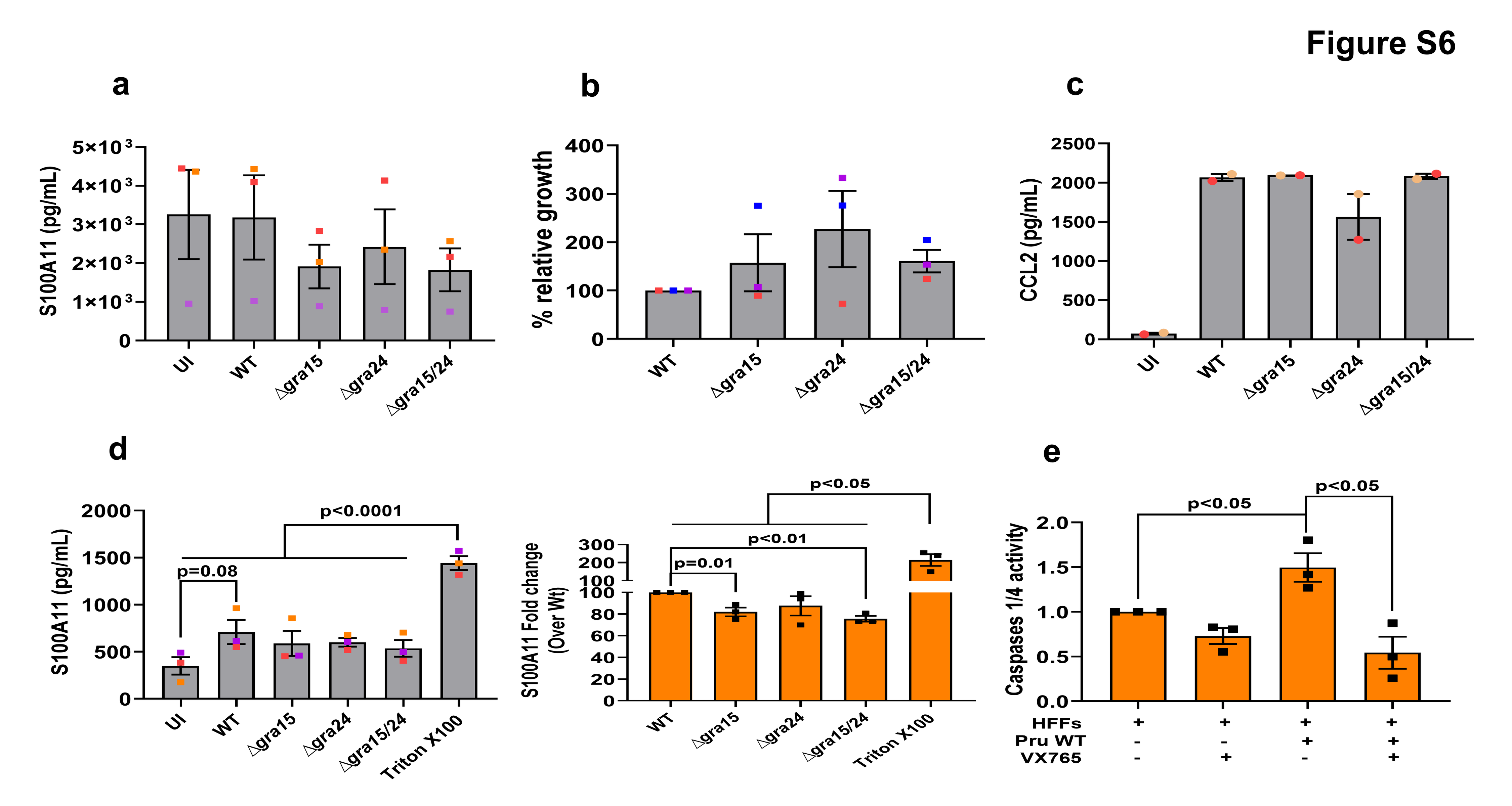

Supplement: S6 Fig — PBMCs or HFFs were infected with indicated Toxoplasma strains at three different MOIs for 24 h, after which supernatants were collected to measure S100A11 in PBMCs (a) and the PBMC lysates were used to measure parasite growth (b). CCL2 was measured from culture supernatants of PBMCs infected with indicated strains as described above (c). S100A11 was measured in HFFs (d). Caspase 1/4 activity assay was measured from HFFs as described in materials and methods (e). Each dot represents the mean value of 3 technical replicates performed for each experiment. Statistical analysis was performed by One-way ANOVA followed by Tukey’s multiple comparison test. Data are represented as mean ± standard error of the mean (SEM). (TIF) [file ppat.1008586.s006.tif]
