# Supplementary material for: Toxoplasma GRA15 and GRA24 are important activators of the host innate immune response in the absence of TLR11
Source: PLoS Pathog. 2020 May 26;16(5):e1008586. doi: 10.1371/journal.ppat.1008586 (PMC7274473; doi:10.1371/journal.ppat.1008586)
Supplement: Attachment — Submitted filename: Rebuttal.docx [file ppat.1008586.s007.docx]

We thank all reviewers and the editor for their suggestions on how to improve our manuscript. Please see our response to each of the concerns below in blue. In addition, in our revised manuscript we have added the ‘Ethics Statement’ subsection and also removed the ‘Data not shown’ phrase from the manuscript.

**Part I - Summary**
Please use this section to discuss strengths/weaknesses of study, novelty/significance, general execution and scholarship.

Reviewer #1: In mice, tlr11-mediated responses are of uttermost importance in the response against the parasite, meaning that more subtle contributions of other pathways are often masked by tlr11. This is circumvented using tlr11 knockout animals. Here, the authors compare WT and Δgra15/24 parasites regarding the immune responses elicited in tlr11 knockout mice, and apply the knowledge gained to systems containing human cells, who do not express tlr11.

In mice, when profilin responses are taken out of the system, GRA15 and GRA24 responses become clearer, but still of secondary relevance when compared to the responses mediated by ROP18 and ROP5. This limits the use of tlr11 knockout mice as a tool to compare mice and humans in this infection model, as IRGs play a major role in mice and not in humans. This limitation is, however, acknowledged in the manuscript, and the conclusions taken from the experiments in tlr11 ko mice are largely validated in human cells, most notably PBMCs.

Here, data points to an essential role of both GRA15 and GRA24 in eliciting IL-1, IL-12, and consequently IFN-g, in PBMCs, linking those cytokines to their transcriptional factors in both models. The study sheds light on the innate immune response elicited by the host upon Toxoplasma gondii infection, enhancing our understanding on how GRA15 and GRA24 trigger an immune response in humans.

Reviewer #2: The following manuscript brought strong arguments that complete previously published data on how GRA15 teams up with the exported effector GRA24 to induce IL-12, TNFα and IL-1β through their ability to activate the NFκB and p38alpha MAPK pathways. TLR11/12 have been reported to mediate important responses for the mitigation of T. gondii infection (Yarovinsky et al., 2005; Koblansky et al., 2013). TLR11- and TLR12-deficient mice were reported to have an impaired IL-12 response to T. gondii linked to the ability to sense the parasite-derived profilin (Yarovinsky et al., 2005; Andrade et al., 2013; Plattner et al., 2008). The authors reported here that in Tlr11-/- mice and in human cells, which do not have TLR11, the T. gondii GRA15 and GRA24 effectors play an important role in induction of IL-12, IL-18 and IL-1β, and thus in the subsequent protective IFNγ secretion. Overall, the data is of high quality, with good controls and the manuscript is well written.

We thank the reviewers for the appreciation of our work and claims in the manuscript. We have addressed all the concerns below.

**Part II – Major Issues: Key Experiments Required for Acceptance**

Reviewer #1: 1) The authors presented data throughout the paper using technical replicates. Whilst I don’t think this is necessarily wrong, I suggest the authors to make it explicit (in the figure legends) the number of independent experiments done in each case. If no other biological replicate was performed, this should also be written unambiguously.

We apologize for the apparent confusion that our display of the data generated. However, all the data presented in the manuscript was obtained from 3 biological replicates. For all IFA and cytokine experiments each dot represents the experimental **mean** of either 15-25 cells (for IFAs) or 3 technical replicates (for ELISAs), as clearly described in the figure legends of each figure. Thus, the presence of 3 dots means that we performed those experiments three independent times or that the data was from 3 mice. This was indicated, for instance, in lines 168-169, 174-175, 255-256, 259, 300-301, 333-334, 377-378 and 383-384.

2) Quantification of c-Rel, p65, and MAPK translocation (figures 1, 3, S1, S3, S5) does agree with the ELISA data, and tell an interesting story. I do, however, have some concerns regarding the data, making it, in my view, necessary to perform the analysis again.
- The quantitation performed in the translocation experiments used an extremely small number of cells. Variability at the single-cell level is notoriously high, as are the variabilities in the quantification of biological data from microscopic images. I recommend increasing the number of cells quantified in each group. Similar experiments in the literature usually perform this analysis in >100 randomly selected cells per treatment.
- The nuclear fluorescence/total fluorescence ratio (or perhaps the ratio between nuclear fluorescence/cytoplasmic fluorescence) would be more accurate, as this would take into account factors such as differences in fluorescence intensity between different samples, the expression levels of c-Rel/p65 in individual cells, etc.

Again, we apologize for the misunderstanding and making the reviewer belief that we measured only 15 cells per group for the IFA and that we performed the statistics and drew the conclusions based on that. Please note that for IFA analysis, we measured in each experiment 15-25 cells per group (Figure 1e left panel graph) and we performed the experiments 3 independent times as indicated in the right panel graph in Figure 1e. We performed the statistical analysis based on the three means from the three independent experiments. We have clearly mentioned in the figure legend that we performed N=3 (lines 174-175). We completely agree that variability at the single level could be high and the left panel graph of Figure 1e indeed shows that. In many manuscripts, authors measure >100 cells in a single experiment and perform statistical analysis based on those measurements, however we feel that rather than doing statistics based on many cells but only done once (N=1), which is not a robust analysis in our opinion, we should conduct 3 independent experiments (N=3) and perform the statistical analysis by comparing the mean values of each group from each experiment, as can be seen in Fig 1e right panel, Figure 3 etc. We clearly mentioned in the figure legend that experiments were done 3 times. Because data of three independent experiments measuring 15-25 cells in each experiment was already significant, we don’t believe that redoing this experiment and measuring more cells will change the conclusions we can draw from this data (even though the data might be even more significant).

We agree with this reviewer that the nuclear fluorescence/total fluorescence ratio would be more accurate, as it would take into account expression levels etc. However, the main conclusions we drew from these figures are unlikely to change. Given that currently our lab is shut down because of the COVID-19 situation and the fact that redoing these experiments will not change the manuscript significantly, we hope that this reviewer will find our quantification of nuclear translocation sufficient.

It should be noted that the ability of GRA15 to activate the nuclear translocation of p65/c-Rel was clearly shown by our group in previous publications (Rosowski et al. 2011, PMID: 21199955, Yang et al. 2013, PMID: 23837824, Melo et al. 2013, PMID: 24367253) and also by other groups (Gov et al. 2013, PMID: 23839215). To take into account the comments by this reviewer and the second reviewer on possible effects of GRA15/GRA24 on inducing the transcription of some of the proteins we quantified (p65, cREL, p38 MAPK), we have added additional sentences in the discussion section (lines 417-424).

3) Even though Alarmin levels were similar across the board, I wonder whether the effects of GRA15 and/or GRA24 on other pathways could still influence CCL2 production in PBMCs. Performing CCL2 elisas could further strengthen conclusions on this topic.

In the revised manuscript we have incorporated the CCL2 ELISA data from human PBMCs infected with wild-type and all the knockout parasite strains used in the study (N=2). As can be seen from the data, the infection with *Toxoplasma* induced a very large amount of CCL2 (Figure S6c), which is in accordance with the recent elegant study by Safronova et al. 2019 (PMID: 30455460). However, we did not observe any influence of either GRA15 or GRA24 on the secretion of CCL2 (Figure S6c and lines 362-364).

Reviewer #2: The images (Fig. 1e) of c-REL translocation are not very convincing. Authors are encouraged to submit more compelling images, if possible. Is also c-REL protein induced by T. gondii infection (as the pictures in Fig. 1e would tend to show)? therefore, is the effect mediated by GRA15 and GRA24 on (only) c-REL translocation (what the authors measure) or likely on its expression; in this regards, c-REL, unlike p65, would belong to the second wave of genes regulated by T. gondii infection. This could change the model of how GRA15 and GRA24 team up, knowing that they have distinct location in the infected cell and likely different kinetics of secretion.

We agree with the reviewer that the images shown in Figure 1e are not as clear as the other images. However, this is due to the nature of the cell type itself, as Raw 264.7 macrophages are not very well suited for IFA. We also performed in parallel the staining in other cell types (HFFs), as can be seen in Figure S3. Nevertheless, following the reviewer’s suggestion, we have incorporated new representative images for c-Rel in Raw macrophages (Figure 1e). We agree with this reviewer that GRA24 and GRA15 could also have an effect on c-REL expression levels and we added some sentences to the discussion that mention this.

**Part III – Minor Issues: Editorial and Data Presentation Modifications**
Please use this section for editorial suggestions as well as relatively minor modifications of existing data that would enhance clarity.


Reviewer #1: Regarding the translocation experiments, please include in the methodology information on how the quantification was performed (software, plugins, etc.).

In the revised manuscript, we have now provided the details of software and camera for nuclear translocation experiments, lines 608-612.

On a cosmetic note, it is hard to visualise some of the translocation events presented, due overlap of blue (which looks saturated in some figures) and red. Addition of the isolated red and blue channels would help.

We agree with the reviewer that some IFA pictures (especially Figure S5), have excessive blue saturation (Hoechst staining for nuclei). Hence, in the revised manuscript, we have provided each individual channel for each part of Figure S5 to clearly show the nuclear translocation event of p65, p38 MAPK and c-Rel. We also wish to point out that this saturation of Hoechst staining is due to the high background which happens only in the case of macrophages (Figure S5 comprise THP1 macrophages), which we feel is a technical difficulty of doing IFAs on these cells. This problem is visible in other papers in the field: for instance, Olias et al. 2016, PMID: 27414498; Cabral et al. 2018, PMID: 30177926. Unlike HFFs, differentiated macrophages are not 100% confluent on coverslips, and after repeated washing during IFAs, all the empty spaces were taken by Hoechst staining, hence the high background. Despite this hindrance, we did not find difficulties in identifying infected cells *versus* uninfected cells.

Some of the ELISA data presented in the article (notably, IL-12 in SF4-a) shows quantification of really small amounts of cytokines. Could the authors amend the methods section to make it more clear which ELISA kits were used for each cytokine?

All the cytokine ELISAs were purchased from Invitrogen except for S100A11 which was purchased from RayBiotech and murine IL18 which was from Sino Biologicals. These are mentioned in lines 576-579, 629-632 and 645-646.

Reviewer #2: 1- TLR11 and TLR12 both contribute to IL-12 production by macrophages, but also conventional DCs and pDCs (Raetz et al., 2013; Koblansky et al., 2013). A recent paper described how IL-12 and IL-2 responses of Toxoplasma-challenged DCs were modulated in a GRA24-dependent fashion (Ten Hoeve AL et al., Front Cell Infect Microbiol. 2019, PMID: 31681626). These data should be discussed knowing the major role DCs are playing in response to T. gondii infections and specifically IL-12 secretion in vivo.

In the revised manuscript, we have discussed the data and added the reference of Ten Hoeve AL et al., 2019. Lines 422-424.

2- Lines 212-216: the authors showed that Tlr11-/- mice infected by i.p. route with 10 cysts of Toxoplasma succumbed to acute toxoplasmosis regardless of the genotype of the strains, refuting previous studies showing that Tlr11-deficient mice can survive i.p. infection with tissue cysts (Koblansky AA et al. 2013; Raetz M et al., 2013; Sturge CR et al. 2013). IP delivery of cysts does not reflect the natural route of infection which is oral ingestion of cysts. It is quite arduous to control the effective load of infectious cysts when these are injected by the IP route. Therefore, these data can be removed to keep only infections with tachyzoite, recognized as relevant in the community.

We believe that keeping the cyst IP infection data is important, as previous studies using *tlr*11^-/-^ mice have performed i.p infections with 20 cysts of ME49 and mostly showed very low mortality rates, which we did not observe here (all the mice succumbed even with cyst infection). This is discussed in lines 438-445. It should also be noted that in the most recent paper from Yarovinsky lab (PLoS Pathog. 2019 Jun 13;15(6):e1007872) high mortality (~75%) after i.p. infection with 20 ME49 cysts was also observed, consistent with our results. Therefore, we prefer to leave this data, so that readers can compare the virulence difference of parasite strains or different mice colony.

3- The IL12/IL23p40 production is as reported here mainly controlled by GRA15 as a consequence of activation of both NF-kB p65 and c-REL subunits by GRA15, while GRA24 exclusively activates c-REL. T. gondii is also able to counteract the NF-kB pathway using exported effector(s) such as TEEGR (Braun L, et al. Nature Microbiology, 2019, PMID: 31036909). The discussion must be widened to integrate this important component, especially when we know the peculiar role of TEEGR that selectively represses the transcription of a subset of NF-κB-regulated cytokines (IL-1β, IL-6, IL-23A and IL-15) and chemokines (IL-8 and CCL20) without altering the expression of other NF-κB-regulated cytokines (IL-12 and IL-18).

In the revised manuscript, we discussed the possible effect of TEEGR on cytokine secretion from human PBMCs in lines 482-487.
